# TelePi: an affordable telepathology microscope camera system anyone can build and use

**DOI:** 10.1007/s00428-023-03685-5

**Published:** 2023-11-07

**Authors:** Almoatazbellah Youssef, Andreas Rosenwald, Mathias Tillmann Rosenfeldt

**Affiliations:** Institute of Pathology and Comprehensive Cancer Centre Mainfranken, Julius Maximilian University of Würzburg, Josef-Schneider-Str. 2, 97080 Würzburg, Germany

**Keywords:** Raspberry Pi, Digital pathology, Telepathology, Open source, Low cost, Low-resource setting

## Abstract

Telepathology facilitates histological diagnoses through sharing expertise between pathologists. However, the associated costs are high and frequently prohibitive, especially in low-resource settings, where telepathology would paradoxically be of paramount importance due to a paucity of pathologists.

We have constructed a telepathology system (TelePi) with a budget of < €120 using the small, single-board computer Raspberry Pi Zero and its High-Quality Camera Module in conjunction with a standard microscope and open-source software. The system requires no maintenance costs or service contracts, has a small footprint, can be moved and shared across several microscopes, and is independent from other computer operating systems. TelePi uses a responsive and high-resolution web-based live stream which allows remote consultation between two or more locations. TelePi can serve as a telepathology system for remote diagnostics of frozen sections. Additionally, it can be used as a standard microscope camera for teaching of medical students and for basic research. The quality of the TelePi system compared favorable to a commercially available telepathology system that exceed its cost by more than 125-fold. Additionally, still images are of publication quality equal to that of a whole slide scanner that costs 800 times more.

In summary, TelePi is an affordable, versatile, and inexpensive camera system that potentially enables telepathology in low-resource settings without sacrificing image quality.

**Supplementary information:**

The online version contains supplementary material available at 10.1007/s00428-023-03685-5.

## Introduction

The number of pathologists is declining, while the case load and complexity are on the rise. Taking the USA as an example, the number of pathologists between 2007 and 2017 dropped by 17.5%, while new cancer cases increased by 41.73% within the same time [1]. To further complicate matters, in the same period, applications for pathology residency training positions dropped by 27.5% [2]. The number of inhabitants per pathologist in the USA is 25,325, while in Germany it is 47,989 [3]. In the African continent, this drops to one pathologist per 784,313; 1,555,555; and 2,533,333 inhabitants in Sudan, Uganda, and Tanzania, respectively [4]. It is inevitable that the field will further progress into subspecialization [5, 6], making expertise more focused, but also more difficult to reach. However, the transfer of knowledge between experienced and junior pathologists is essential to the practice of pathology [7].

Pathology traditionally depends on the visualization of tissue samples on glass slides using brightfield microscopy. Digitization transforms the way pathologists work [8]. This can be in the form of whole slide imaging (WSI), which can be used for routine clinical diagnostics as well as a wide range of other purposes or more focused for remote consultations as in telepathology [9]. The College of American Pathologists define telepathology as “the practice of pathology and cytology in which digitized or analog video, still image(s), or other data files are examined, and an interpretation is rendered that is included in a formal diagnostic report in the patient record” [10]. At its core, telepathology requires a digital camera system to capture the image and an infrastructure to distribute and visualize the images afar [11]. Not only can telepathology facilitate the primary diagnosis of samples, but also improve the teaching of junior pathologists by more experienced staff, as well as in basic research, student education, and remote consultations [12–15].

Cost is a significant hurdle when establishing a telepathology system, especially in a limited-resource setting, where they are needed the most [14, 16, 17]. A telepathology system based on WSI requires an initial high investment to acquire slide scanners, followed by substantial running costs, including service, data storage, internet bandwidth, and computer infrastructure. The cost and maintenance of a WSI system was estimated to be more than the allocated budget to most private or teaching hospitals in underdeveloped countries [16]. A professional telepathology system with camera and software [10] requires a significant financial investment that often includes annual licensing fees.

Here we describe the assembly and implementation of TelePi: an affordable, high quality telepathology system using off-the-shelf hardware parts and open-source software. The Raspberry Pi (RPi) (https://www.raspberrypi.com/) is a low-cost, credit card–sized, single-board computer (SBC) which can run a complete Linux operating system (OS).

The RPi is modular and open in design. Different camera modules can be directly connected to it. Here, we chose the High Quality Camera Module (HQ Camera) [18]. There are several software libraries which can be used to control the camera functions and create live streams of the video output of the camera. The open-source nature of the software ensures that modifications and improvements can be openly shared with the wider community without worrying about copyright restrictions.

TelePi costs less than €120 and can be used as a telepathology system or as a high-quality microscope camera. This work facilitates the use of open standards and royalty-free components to promote open pathology and the adoption of telepathology in low-resource settings.

## Materials and methods

**Fig. 1:**
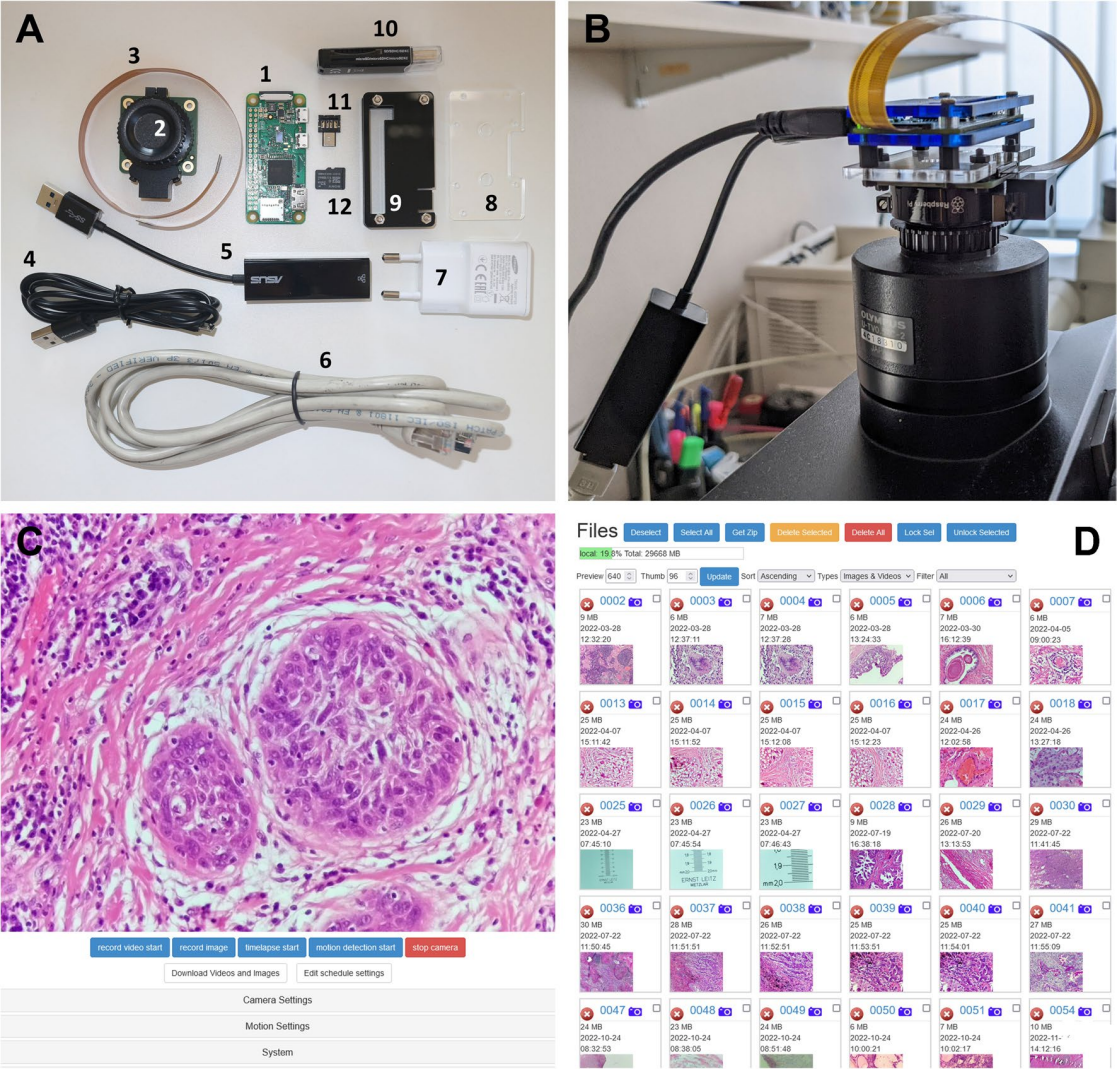
Overview of the TelePi system. **A** Required components for the hardware assembly: (1) RPi Zero, (2) HQ Camera, (3) flex ribbon cable, (4) Micro-USB cable, (5) USB to Ethernet adapter, (6) Ethernet cable, (7) USB power adapter, (8) mounting bracelet, (9) acrylic case, (10) microSD card reader, (11) Micro-USB to USB A adapter, (12) microSD card. **B** Assembled and running TelePi. **C** The RPi Cam Web Interface showing a live image. In the bottom are the controls for the camera functions. **D** Image and Video Download interface

A step-by-step software installation and configuration guide is available online (https://dx.doi.org/10.17504/protocols.io.3byl4jp22lo5/v1). Hardware costs at the time of writing are detailed in Table 1. Figure 1A illustrates the individual components of the system.

**Table 1:** Estimated costs of materials for TelePi based on current market prices in Germany (in Euros)

Item type	Item	Cost (€)
Hardware	RPi Zero v1.3	12
	RPi HQ Camera	60
	Flex ribbon cable for RPi Zero	4
	USB to Ethernet adapter*	13
	Micro-USB to USB adapter*	2
	Micro-USB charging cable and USB charger*	5
	32 GB microSD card	6
	Mounting bracket and enclosure	6
Software	Raspberry Pi OS	0
	RPi Cam Web Interface	0
	Fiji	0

*Parts that often could be recycled from old smartphones/laptops

### The Raspberry Pi Microscope Camera

#### Hardware description

The main parts of TelePi are the RPi Zero v1.3 SBC and the RPi HQ Camera, which has a 12.3-megapixel CMOS image sensor (Type 1/2.3 inch, Sony IMX477-AACK). This camera itself is sold without a lens and has a standard C-mount for attaching lenses. The HQ Camera was previously characterized for use in scientific experiments [19, 20]. The assembled camera system was mounted on Olympus microscopes (BX41 and BX53, Olympus, Tokyo, Japan) using an Olympus 0.5X camera adapter (U-TV0.5XC-2).

#### Hardware assembly

The whole assembly process and the software installation can be done in less than 1 h:

- Fix the HQ Camera to the mounting bracket with the supplied screws then attach the RPi Zero with its enclosure to the other side of the bracket. Connect the camera to the RPi Zero using the ribbon cable.
- Attach the network cable to the USB to Ethernet adapter and connect it to the RPi Zero via the Micro-USB to USB adapter.
- Insert the microSD card in its slot on the RPi Zero (after installation of the software, see below).
- Connect the Micro-USB power cable to power on the system. The system runs headless with no need to attach a monitor, keyboard, or mouse.

The system is portable and can be shared between microscopes with a C-mount camera port. The assembled system is shown in Fig. 1B.

#### Software installation

We used the official RPi OS (https://www.raspberrypi.com/software/), built on Debian 11 (https://www.debian.org/), a free and open-source GNU/Linux distribution. The live camera interface is available after installing the RPi Cam Web Interface (https://elinux.org/RPi-Cam-Web-Interface). It offers a graphical user interface via any web browser to control the camera options, view a video live stream, and record images and videos as well as view already saved ones [21]. Specific user profiles that manage camera permissions can be assigned.

To install the RPi OS on the microSD card:

- On a separate PC (here we are using a Windows PC) download and install the 32-bit version of the RPi OS Lite or Legacy Lite to a microSD card through the official installer (https://downloads.raspberrypi.org/imager/imager_latest.exe). In Advanced Options, enable Secure Shell (SSH) for remote access and add a username and a password for the administrative user.
- Insert the microSD card into the assembled system and power it on. Use PuTTY (https://www.putty.org/) on the Windows PC and initiate a SSH session using the IP address of the RPi Zero.
- In the SSH session, log in using the assigned username and password. Type sudo raspi-config and enable Legacy Camera Support under Advanced Options. Save changes and reboot when prompted to.
- Log in again in PuTTY and run sudo apt update; sudo apt upgrade -y; sudo apt install git -y.
- To install the RPi Cam Interface, run the following command: git clone https://github.com/silvanmelchior/RPi_Cam_Web_Interface.git
- Navigate to the folder containing the installation files by typing cd RPi_Cam_Web_Interface then run./install.sh and follow the on-screen prompts to the end. Add a username and password for the live stream when asked to.
- Using a standard web browser (e.g., Mozilla Firefox: https://www.mozilla.org/en-US/firefox), type http://IP-address-of-RPi/html. Log in using the live stream username and password to access the interface (Fig. 1C, D).

### System and image quality validation

TelePi was tested as a tool for intraoperative consultation between an external grossing laboratory and our main institute between October 2022 and July 2023. The telepathology system linked one pathologist with other staff members through its live stream capability. Standard hematoxylin and eosin (HE)-stained slides of frozen as well as formalin-fixed, paraffin-embedded (FFPE) sections of routine diagnostic material were used. The system was tested against a professional telepathology system with a 5-megapixel USB 3.0–connected camera, which has been used for the last 2 years in our institute. The commercial system was simultaneously mounted on the same microscope (Olympus BX53) as TelePi on an identical 0.5X camera adapter and used according to the manufacturer’s instructions. Its camera displays, after the installation of the required software, a native live view on the PC to which the camera is directly connected to via USB 3.0 and broadcasts a live stream through the internet for external users.

To assess image quality of TelePi as a static microscope camera, a commercial WSI scanner and a professional-grade, 5-megapixel microscope camera were used to capture the same regions of interests (ROIs).

### Additional configuration steps

In the detailed protocol (https://dx.doi.org/10.17504/protocols.io.3byl4jp22lo5/v1), it is shown how to extract further functions. These include:

- Mounting a network drive containing all recorded images/videos on a Windows PC for quick access and export of saved image and video files
- Adding scale bars to the captured images using calibration data in Fiji [22]
- Stitching of overlapping images to create a larger image using Fiji [23] (A dataset is provided as an example at https://doi.org/10.5281/zenodo.8151968.).

## Results

### Remote access

The live web interface (Fig. 1C) was accessed simultaneously and run in parallel multiple times to stress test the system with up to four different streams on the same network, also within different physical locations and networks using a virtual private network (VPN). No system or network errors occurred.

### Image quality

**Fig. 2:**
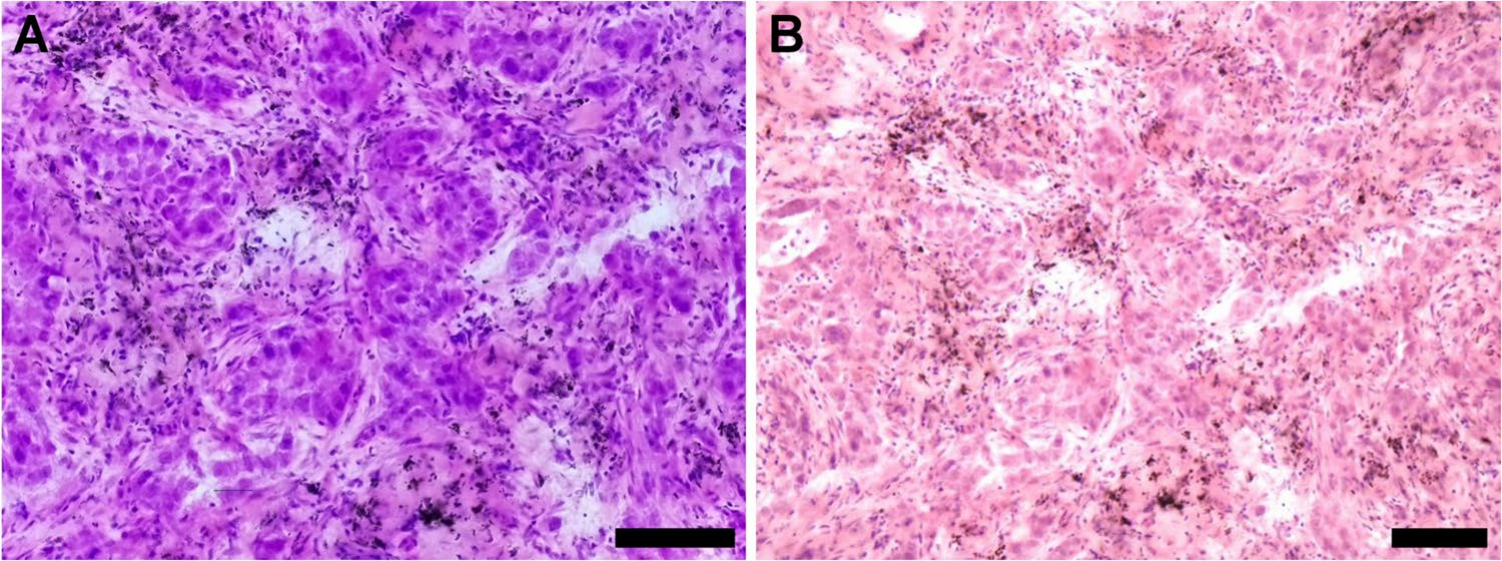
Image quality comparison between TelePi and the commercial telepathology system using a frozen section from a lung cancer specimen (HE). **A** TelePi live image. **B** Commercial system live image. Both images are taken simultaneously on the same microscope with the same 20X objective. Scale bars are 100 µm

We tested two different image modes. The live image mode showed a live stream of the microscope slide moved on the microscope with a maximum resolution of up to 1440 × 1080 pixels (we opted for 1024 × 786 to balance fluid motion and high resolution on the RPi Zero) (Fig. 2A; Supplementary Video 1 upper left corner). Static mode has a resolution of up to 4056 × 3040 pixels (Supplementary Fig. 1) and captures images that can be viewed through the web interface or exported to Fiji (Fig. 1D).

It is possible to capture video recordings with a maximum resolution up to 1920 × 1080 pixels (we used 1280 × 960 for a more optimal 4:3 aspect ratio instead of 16:9) (Supplementary Video 2). This video mode is not shown live; rather, it is written directly to the microSD card and needs time to be encoded by the RPi Zero. The upper left corner of Supplementary Video 1 shows the same sequence as in Supplementary Video 2 but through the live mode as shown in a web browser window instead of direct recording.

**Fig. 3:**
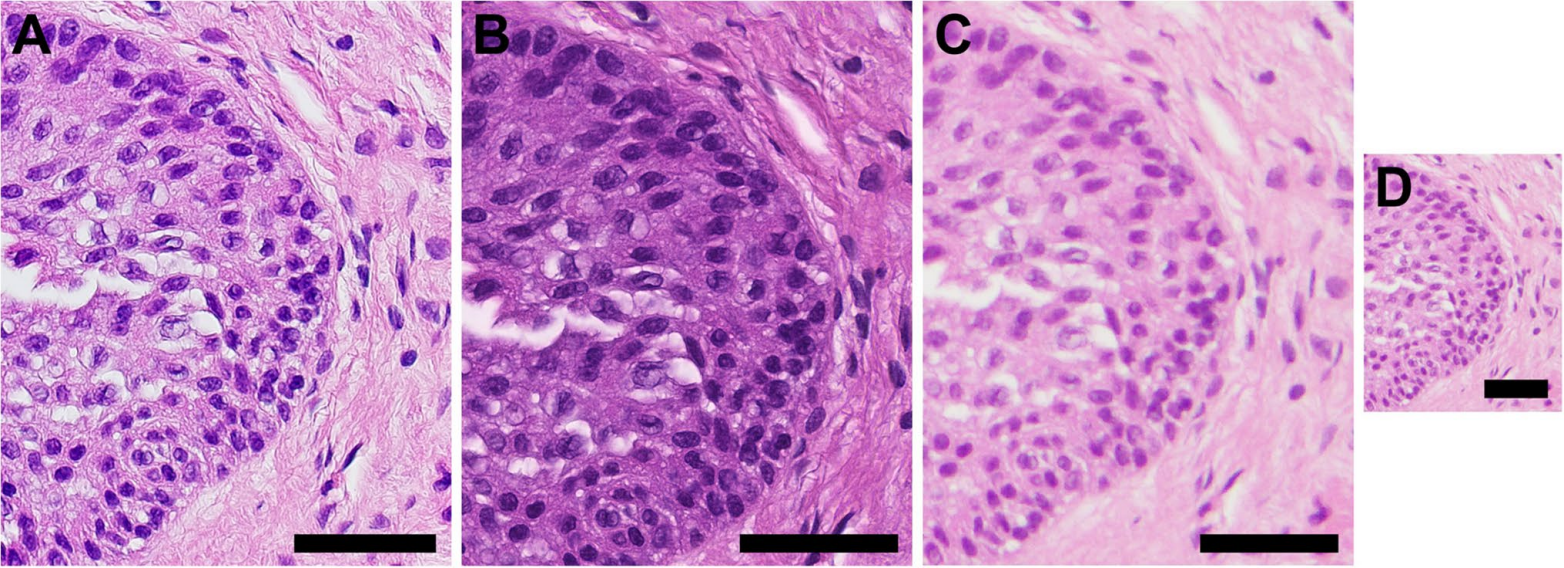
Comparison of TelePi static image quality to commercial systems using FFPE material from a ureter sample (HE). **A** A cropped segment from a TelePi full resolution static image (20X). **B** Same ROI cut from a commercial-grade WSI scanner in 40X. **C** The same ROI captured by a commercial digital microscope camera (20X). Due to the lower resolution of the image from the commercial camera, it had to be magnified to be of the same size as in A and B, with resulting pixelation. **D** Same ROI as in C from the commercial camera but presented in the original image size. Scale bars are 50 µm

The image quality of static images from TelePi (Fig. 3A) was comparable to that of the same ROIs taken with a WSI scanner (Fig. 3B) and a commercial microscope camera (Fig. 3C, D). Using a Fiji plugin [23], overlapping static images taken via TelePi were stitched to form one larger image in Supplementary Fig. 2A. The same ROI was cropped from a WSI scanner and is displayed in Supplementary Fig. 2B. The image quality of TelePi shows almost no quality loss; however, there is at least an 800-fold price difference between both systems.

### Performance as a telepathology system

We used the same slide on the same microscope to simultaneously capture the streaming images using TelePi (Fig. 2A) and the commercial system (Fig. 2B). The static image from TelePi (Supplementary Fig. 1A), as well as the original view from the commercial system (as seen on the sender’s PC) (Supplementary Fig. 1B), illustrated the maximum resolution of their respective cameras.

The live image was sharp, responsive, and adequate for performing routine remote consultations on frozen section material. When the observation of finer cellular morphology or nuclear details was needed, a full resolution static image could be captured on TelePi (by either user) and then viewed using the same web interface (also by either users) (Supplementary Fig. 1A**)**. This was not possible on the commercial telepathology system, and the transmitting pathologist sees an image of much better quality (Supplementary Fig. 1B) than the receiving pathologist (Fig. 2B). With TelePi, both always get the same image quality via live streaming.

In comparison to the commercial system, the slide motion in live view using TelePi was smoother and sharper. There was negligible delay between real movement of the slide and that on the live stream of TelePi, while that of the commercial camera was noticeably delayed with skipping, so that the movement path could not be clearly followed (Supplementary Video 1).

Figure 2 and Supplementary Fig. 1 show the field of view differences between TelePi and the commercial system. The sensor size in the RPi HQ Camera is 1/2.3 inch, while that of the commercial camera is 2/3 inch, i.e. the sensor of TelePi is 35% smaller.

TelePi displays more saturated colors compared to the commercial telepathology system or the webcam in Supplementary Video 1. White balance settings were calibrated on the commercial system per manufacturer’s instructions before imaging. TelePi had white balance settings set to the preset “Flash.” The color saturation when observed directly by the pathologist from the eyepiece is similar to the one displayed by TelePi.

A summary of the comparison between TelePi and commercial systems is summarized in Table 2.

**Table 2:** Comparison between TelePi, a commercial telepathology camera, a whole slide scanner, and a standard brightfield microscope camera. N/A, not applicable

		TelePi	Commercial telepathology system	Whole slide scanner	Brightfield microscope camera
Live view	Shareable live view	Yes	Yes	No	No
	Live view responsiveness	Negligible lag	Considerable lag	N/A	N/A
	Live view customizations	Yes	Yes	N/A	N/A
Static image and video capture	Capture static images	Yes (by both sides)	Yes (only by transmitting side)	Yes	Yes
	Shared real-time static images	Yes	No	No	No
	Video capture	Yes	No	No	No
System requirements	Software requirements	Open source	Proprietary	Proprietary	Proprietary
	Hardware requirements	Standalone system	Additional PC	Additional PC	Additional PC
Cost	Hardware cost (estimated)	€120	€15,000	€100,000	€5,000
	Additional costs	No	Yes (Recurring license fees)	Yes (Software updates)	Yes (Recurring license fees)
Usage	System portability	Can be shared across multiple microscopes	Bound to one PC	Bound to one PC	Bound to one PC
	Hardware repair	Do-it-yourself with off-the-shelf parts	Through manufacturer	Through manufacturer	Through manufacturer

## Discussion

The introduction of telepathology opened new horizons for collaboration, education, and research [11, 13] in a discipline that remains since its inception bound by a compulsory use of glass slides. However, this switch is not without its difficulties, mostly due to significant costs [16].

Here we introduced a telepathology camera system, TelePi, that can be self-built, modified, and operated. We compared TelePi to a professional system that meets the In Vitro Diagnostic Regulations (IVDR) for medical devices in the European Union and has a CE-Label. TelePi costs almost €120 while the commercial system initially costs almost €15,000, with an annual software license fee of €2,000. Our setup does not require licensing. TelePi runs independently from other computer hardware and software, and it will not cease functioning because the user updated their drivers or their OS.

The quality of TelePi was equal and in many instances superior to that of the commercial telepathology system. The live stream is accessible on any web browser and on any device connected to the same network. Through the capture of static images at full resolution of the HQ Camera sensor, fine cytological details can be visualized that are not clearly visible in the live stream.

In comparison, the commercial system showed a significant output lag for the receiver only, which made communication between the transmitting pathologist and the receiver harder. With TelePi, there is no quality or speed difference, and all users see exactly the same image. Using the commercial system, image artifacts often prevented fine cytomorphological evaluation, and there was no option to directly share static, high-resolution images in real time. This problem is mitigated in TelePi, as both sides view the same live stream and can take full resolution still images or videos.

Our system can also be directly used as a high-quality brightfield microscopy camera and, through Fiji, can be expanded to deliver stitched images and thereby a whole slide image. Using Fiji is not unique to this system but illustrates the power of using open-source software to provide functionalities which are available in commercial systems for an added cost or a continuous licensing fee.

While TelePi can be left operating in permanent live streaming mode, it is more suitable for performing telepathology or imaging individual cases and not for high throughput activities. It was not designed for digitizing the whole pathology workflow or for the extensive archival of cases. One caveat that might prevent the use of TelePi as a telepathology system in developed countries is that it has not received regulatory approval. To achieve this, it would require the commercialization of TelePi along with a significant financial investment. It is the desire of the authors to leave the system open and accessible. Moreover, in low-resource settings, we believe that the potential clinical benefits of TelePi outweigh its regulatory limitations.

The use of video messaging applications to transmit a live video stream through a smartphone mounted on the eyepiece of the microscope has been described [24–27]. Others captured still images using smartphones and shared them to external observers using emails and messenger applications [28, 29]. Live microscope images have also been shared using common video conferencing applications [30–32]. These methods do not usually conform to patient privacy and data safety guidelines. TelePi can run within a firewalled network and is not accessible externally except through a VPN or via port forwarding. The transmitted and/or recorded images are completely anonymous, and password-protected user accounts with restrictive permissions can be assigned.

Other camera systems are marketed for teaching but not as medical devices, are relatively cheap with a cost of less than €2000, and could potentially function as telepathology systems. These are either limited by proprietary software, lack of IVDR certification, reliance on wireless connectivity that might raise security concerns for the IT departments, and are less versatile than TelePi.

To the best of our knowledge, this is the first report to use a RPi with its HQ Camera as a live stream microscope camera for a telepathology application by directly mounting the HQ Camera via its C-mount on a trinocular brightfield microscope and relying solely on RPi hardware. Previous work used older RPi camera modules mounted on microscopes for teaching purposes by displaying the image directly on a physically connected monitor [33] without live video streaming and relied on 3D-printed converters to mount the RPi camera on the microscope’s eyepiece part. Others connected a USB webcam to the RPi SBC and created a live video stream for telecytology [34] but experienced a significant transmission lag (around 6 s). The camera was also connected through custom-made parts to the microscope without standard camera mounting systems. There were two reports of end users using the HQ Camera as a microscope camera using the C-mounting system, but both only displayed the captured video on a physically connected monitor. Moreover, the microscopes used were simpler, hobbyist microscopes for examination of integrated circuits [35], or studying microorganisms in water samples [36].

## Conclusion

Here we described a detailed protocol to self-assemble a microscope camera using off-the-shelf parts and to install the necessary open-source software to turn it into a fully capable telepathology system (TelePi), for approximately €120. The system is stand-alone and royalty-free. Running costs are essentially non-existent and advanced computer knowledge is not needed. Operating TelePi can be confined to restricted privacy settings. The low cost and wide availability of parts can solve an important problem in the wider adoption of telepathology: cost. This can enable better training of junior pathologists by more experienced staff as well as provide better diagnostic services using the expertise of subspecialized pathologists for difficult and rare cases, especially in low-resource settings.

### Supplementary Information

Below is the link to the electronic supplementary material.

Supplementary file1 (JPG 2743 KB)

Supplementary file2 (JPG 7619 KB)

Supplementary file3 (DOCX 15 KB)

Supplementary file4 (MP4 21815 KB)

Supplementary file5 (MP4 69181 KB)

## Data Availability

The datasets used and/or analyzed during the current study are available online (https://doi.org/10.5281/zenodo.8151968) or from the corresponding author upon reasonable request. Source code of used software is available on the website for each project.

## Declarations

The use of anonymized human tissue was approved by the local ethics committee of the Julius Maximilian University of Würzburg (approval number 20230612 02).
