# Supplementary figures and images for: TelePi: an affordable telepathology microscope camera system anyone can build and use
Source: Virchows Arch. 2023 Nov 7;485(1):115–22. doi: 10.1007/s00428-023-03685-5 (PMC11271423; doi:10.1007/s00428-023-03685-5)

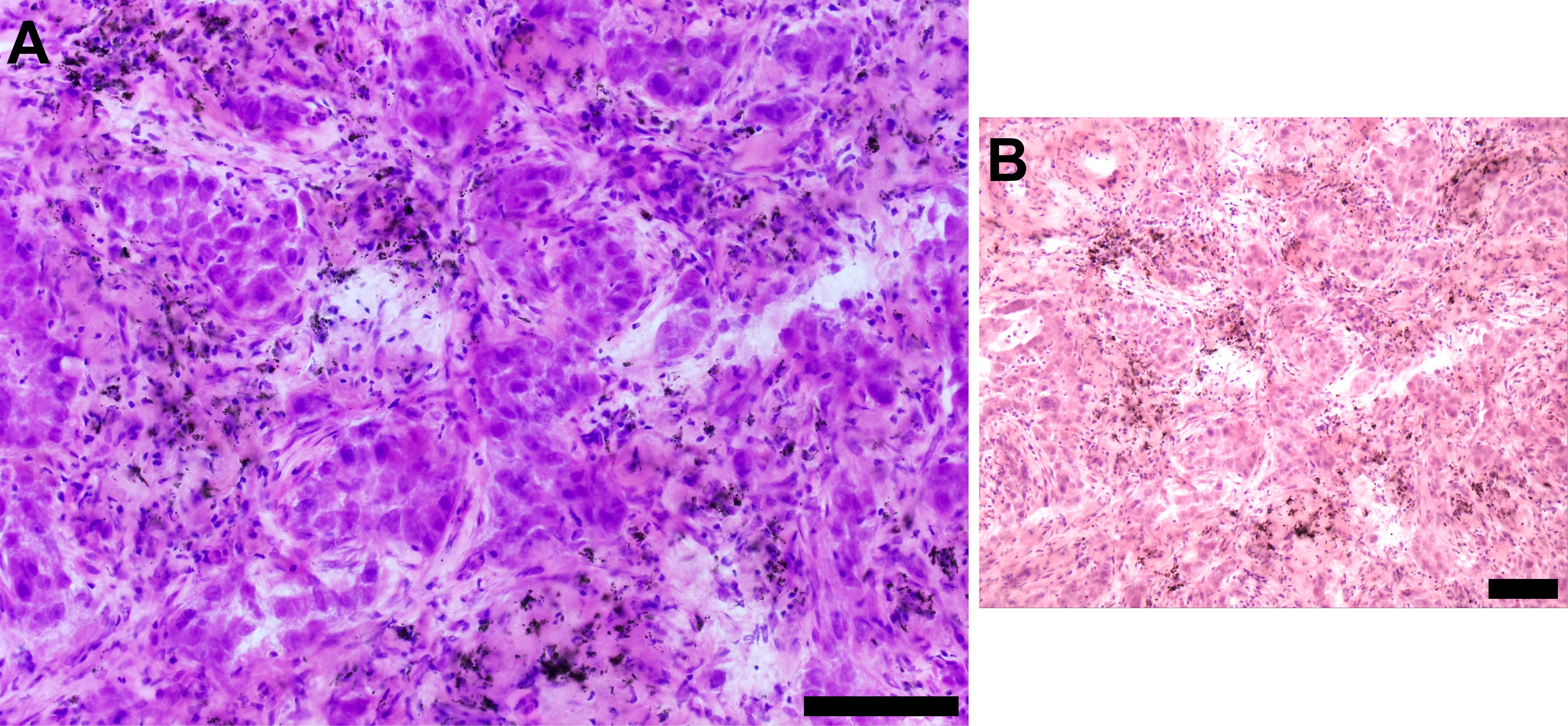

Supplement: Supplementary file 1 — Supplementary file1 (JPG 2743 KB) [file 428_2023_3685_MOESM1_ESM.jpg]

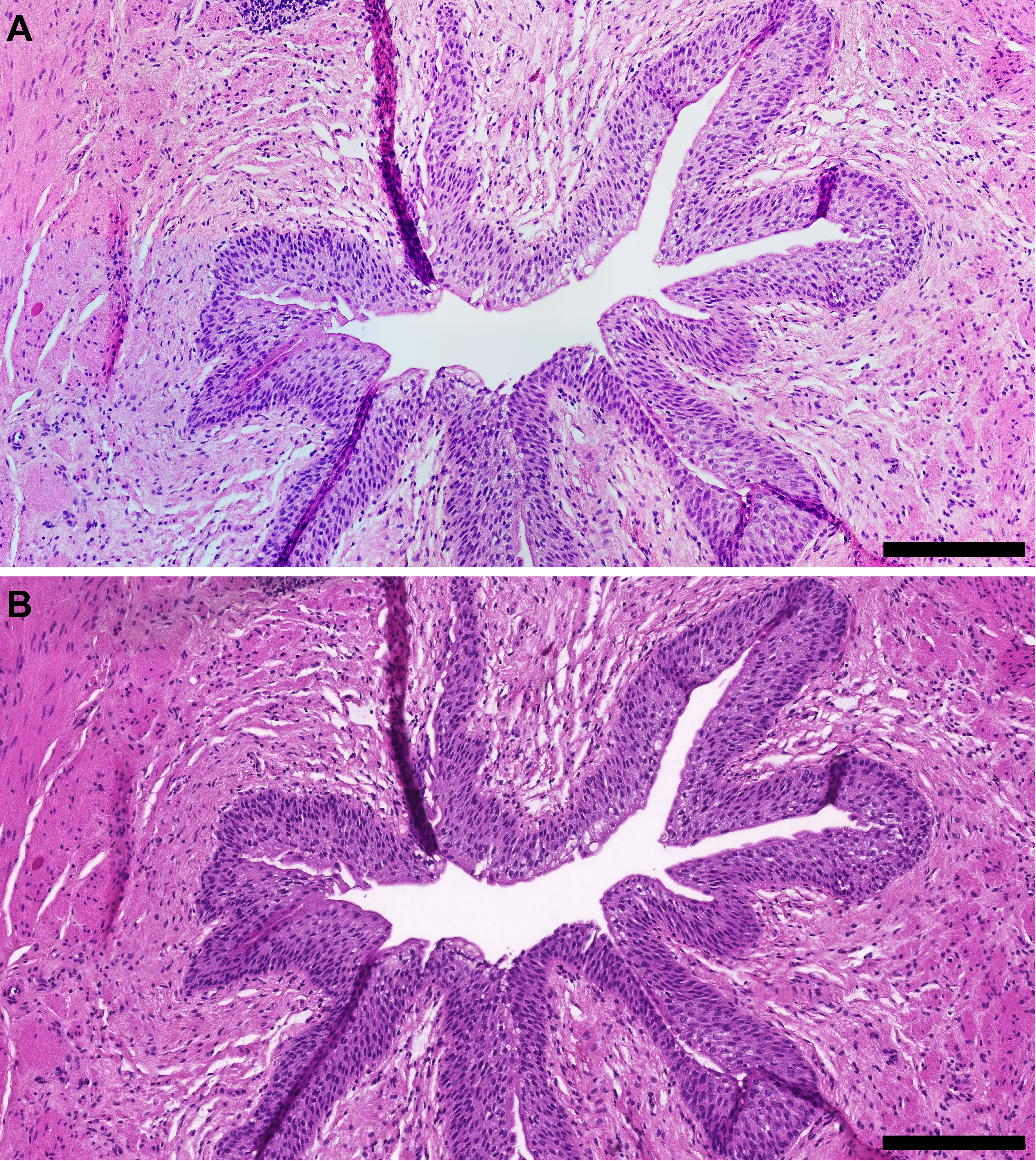

Supplement: Supplementary file 2 — Supplementary file2 (JPG 7619 KB) [file 428_2023_3685_MOESM2_ESM.jpg]
