# Supplementary material for: TelePi: an affordable telepathology microscope camera system anyone can build and use
Source: Virchows Arch. 2023 Nov 7;485(1):115–22. doi: 10.1007/s00428-023-03685-5 (PMC11271423; doi:10.1007/s00428-023-03685-5)
Supplement: Supplementary file 3 — Supplementary file3 (DOCX 15 KB) [file 428_2023_3685_MOESM3_ESM.docx]

**TelePi: An affordable telepathology microscope camera system anyone can build and use**

**Youssef, A., Rosenwald, A., Rosenfeldt, M. T.**

**Supplementary Information – Legends**

**Supplementary Video 1: Video recordings comparing live streams of TelePi to a commercial telepathology system.** The live view of the RPi Cam Web Interface from TelePi is shown in the upper left corner. In the upper right corner is the live view from the commercial telepathology camera. In the bottom half of the frame is direct footage from the eyepiece as seen by the pathologist and captured using a webcam (Logitech HD C615) mounted in front of the eyepiece using a tripod. Live videos were running in separate windows on the same Windows PC in Firefox web browser and were simultaneously captured using OBS Studio (<https://obsproject.com/>). This is the same sequence as the one directly recorded on TelePi and shown in **Supplementary Video 2**. The tissue shown is a HE-stained frozen section of a ureter (20X).

**Supplementary Video 2: Captured video recording from TelePi from the RPi Cam Web Interface (at a resolution of 1280 x 960 pixels) running in parallel to the live stream in Supplementary Video 1.** The video was recorded and encoded on the RPi Zero without any extra steps. This video is not shown live on the RPi Cam Web Interface, rather it is saved directly to the microSD card after being encoded and is of superior quality than the live video. The tissue shown is a HE-stained frozen section of a ureter (20X).

**Supplementary Figure 1: Image quality comparison of static full resolution images between TelePi and a commercial telepathology system.** A) TelePi full resolution static image from the same field of view as in **Figure 2A** (HE, 20X). B) Commercial telepathology system full resolution static image from the same field of view as in **Figure 2B** (HE, 20X). Scale bar are 100 µm.

**Supplementary Figure 2: Image quality comparisons of stitched images taken at 20X using TelePi in static full resolution mode (A) and a WSI scanned at 40X using a commercial scanner (B).** A HE-section from FFPE material with a ureter sample was chosen for imaging. (A) was stitched using the Stitching plugin in Fiji and afterwards cropped, otherwise unchanged. Figure 4B is cropped to represent the same ROI as in (A) using QuPath and ImageJ. Scale bars are 200 µm. The images in this figure were previously published in our online open-access protocol (<https://dx.doi.org/10.17504/protocols.io.3byl4jp22lo5/v1>) on protocols.io under the Creative Commons License CC BY 4.0.
